# Vigilance responding to number of conspecifics among mixed groups of cranes in demilitarized zone

**DOI:** 10.1080/19768354.2018.1453544

**Published:** 2018-03-23

**Authors:** Piotr G. Jabłoński, Sangdon Lee, Elizabeth Ellwood

**Affiliations:** a Department of Biological Sciences, Seoul National University, Seoul, Korea; b Department of Environmental Science and Engineering, Ewha Womans University, College of Engineering, Seoul, Korea; c La Brea Tar Pits and Museum, Natural History Museum of Los Angeles County, Los Angeles, CA, USA

**Keywords:** DMZ, endangered species, group size, *Grus japonensis*, *Grus vipio*

## Abstract

Numerous studies have addressed antipredatory benefits of mixed-species flocks of foragers, but studies on individual's vigilance as a function of group size are limited. In the Cheolwon area of the Korean Demilitarized Zone, vigilance of the subordinate White-naped cranes (*Grus vipio*) in 11 groups composed of conspecifics and the dominant Red-crowned cranes (*Grus japonensis*) was examined. Vigilance correlated negatively with group size due to negative correlation with the number of conspecifics, but not the dominant heterospecifics. This is consistent with the hypothesis that a decrease in vigilance in larger groups is due to antipredatory benefits from increased predator detection in larger groups (associated with the presence of a larger number of conspecifics). This suggested that the mechanism leads to canceling out of the otherwise expected antipredatory benefits to the subordinate species from the increased predator detection by larger group size (associated with larger number of dominants). This is one of only a few behavioral studies of these endangered crane species in the relatively inaccessible wintering area of international importance in the areas of high conservation value.

## Introduction

The question of why some species form interspecific groups has been one of the central issues in behavioral ecology since Hamilton’s (1971) and Pulliam’s (1973) theoretical papers and early empirical studies of gregariousness (e.g. Caraco 1979; Lazarus 1979). Although gregariousness has been intensely studied for more than 30 years (Elgar 1989; Quenette 1990; Bednekoff and Lima 1998; Giraldeau and Caraco 2000; Treves 2000; Beauchamp 2010), more research in this area is needed.

Studies of mixed-species groups have developed parallel to the research on mono-specific groups. They comprise numerous observational (e.g. Hutto 1988; Terborgh 1990; Suhonen 1993; Suhonen et al. 1993; Thiollay and Jullien 1998; Jabłoński and Lee 1999, 2002; Goodale and Kotagama 2008; Sridhar and Shanker 2014), and experimental (e.g. Berner and Grubb 1985; Alatalo and Moreno 1987; Yaukey 1995; Dolby and Grubb 1998; Farine et al. 2012) approaches in natural settings (Diamond 1981; Sridhar et al. 2009; Harrison and Whitehouse 2011) supplemented by modeling of intra-flock behavioral interactions (Thompson and Lendrem 1985). Because mixed-species flocking serves different purposes in different situations, it is necessary to study more species at different locations in order to obtain a proper perspective on the importance of various mechanisms of flocking.

Vigilance of individuals is of pivotal importance in understanding and testing the mechanisms that shape groups and their member's behavior. Theoretical models (Pulliam et al. 1982; Hart and Lendrem 1984; Beauchamp 2013) predict that vigilance should decrease with an increasing group size because other group members may alert an individual about approaching danger or because the risk of predation in a larger group is lower (*antipredatory vigilance* hypothesis). In such situations, increase of vigilance in response to an increase in group's size has been observed in monospecific groups (Knight and Knight 1986). The effect of dominant species on subordinate species’ vigilance in mixed-species flocks can be viewed in a similar manner (Valone and Wheel Barger 1997; Pravosudov and Grubb 1999; Srinivasan and Quader 2012). For example, increased predator detection probability by a group due to an increased group size associated with the increased number of individuals of the dominant species should lead to a decrease in vigilance of the subordinate species. However, the increase in the number of individuals of the dominant species may require more vigilance in the subordinate species to detect the approaching dominants (Popp 1988). The two opposing trends may cancel out or the latter one can be prevalent leading to positive correlation between the number of dominants and vigilance of the subordinate species. Even if the outcome of the two opposing trends is a negative relationship between the number of individuals of the dominant species and the vigilance of the subordinate species, then such an effect should be less pronounced than the effect of the number of conspecifics on the vigilance of the subordinates. This prediction has been examined relatively rarely because most empirical studies have investigated vigilance as a function of presence or absence of the heterospecifics (e.g. Popp 1988; Dolby and Grubb 1998; Kristiansen et al. 2000; Randler 2004; Lee et al. 2007a; Sridhar et al. 2009; Farine et al. 2012). We were able to find relatively few studies on the effect of the number of conspecifics and heterospecifics on focal species vigilance in mixed-species groups of birds (Lazarus and Symonds 1992) and mammals (Fitzgibbon 1990). More data from a variety of mixed-species groups are needed for better understanding of vigilance dynamics in such groups. Because vigilance may affect bird's survival and foraging, understanding of the mechanisms that shape vigilance in natural mixed-species groups of endangered birds in their natural habitat is especially important. Therefore, we decided to address these issues using endangered crane species.

Groups composed of endangered (IUCN category) Red-crowned cranes (*Grus japonensis)* and vulnerable White-naped cranes (*G. vipio*) on their wintering grounds provide good opportunity for studying the above relationships. Wintering cranes are vigilant due to a variety of factors including human disturbance (Pae and Won 1994; Yoo 2004), which may be perceived as danger by birds (Frid and Dill 2002). The Red-crowned crane is larger than the White-naped crane (Won 1981) about 10% more in body size, and it dominates the White-naped crane in interactions involving threat postures and fights. Pae and Won (1994) showed that in 68% of the 56 interactions involving threat postures and supplant behavior, the Red-crowned crane was the dominant species, and in 10 out of 11 fights the Red-crowned crane was the winner. Foraging White-naped cranes often stopped foraging and moved away from approaching Red-crowned cranes (Lee et al. 2007a). Our previous report suggested foraging benefits to the Red-crowned cranes in the presence of the White-naped cranes. Here, we argued that this dominant-subordinate relationship may lead to a lack of negative correlation, or a presence of a positive one, between the number of Red-crowned cranes (the dominant species) and the vigilance of White-naped cranes (the subordinate species) in a two-species group, while such a correlation between vigilance of the subordinate species and the number of conspecifics should be negative in accordance with the antipredatory hypothesis.

The aim of the present research is to study the effect of the group size and group composition on the vigilance of the subordinate species, the White-nape crane, to evaluate the relative importance of ‘*anti-predatory*’ and ‘*anti-dominant*’ functions of subordinate's vigilance to provide behavioral information that may be useful in their conservation.

## Methods

### Observation of cranes

The Cheolwon area located at 38.07–38.45°N, 127.04–127.57°E in the ‘Demilitarized Zone (DMZ)’ between South and North Korea is an important wintering and stop-over site for waterfowl (Lee 2004). Red-crowned cranes (250–300 individuals) and White-naped cranes (200–350 individuals) winter in this area every year (Birdlife International 2001). We conducted observations during the winter, when cranes are present at the study site. Groups of White-naped cranes and Red-crowned cranes were videotaped during five visits to the Cheolwon area in one month between 21 December 2002 and 25 January 2003 during daytime before 5 pm. To avoid disturbance by the observers, the camera and the observer were carefully situated in a car at a distance of 200–300 meters to the focal group of cranes. To avoid bias due to behavioral differences between birds on the edge of the group and those in the middle of the group, we avoided videotaping of birds located on the edge of the group. Data were collected for 11 two-species groups by selecting separate groups in the study areas. Behavior was based on an average 7.5 min. of video-recording/individual (5.5 [1.5–23.8] minutes; median [minimum - maximum]). Preliminary analysis showed no significant relationship between duration of observation of an individual and proportion of vigilance (Spearmen rank correlations: *r_s _*= −0.19, *n *= 60, *P *= 0.20). Therefore, we could use all the observations without risk of bias due to different durations, especially after calculating average vigilance in a group as described below. On average 2.4 (range: 1–5) White-naped cranes were sampled in a group. Categories of behaviors were noted for each bird at 15-second intervals: *Vigilance posture*: the bird's neck is straighten up with its beak often directed horizontally or upward, the bird is still and carefully observing the surroundings; *Remaining behaviors* that comprised resting, locomotion, and comforting (cleaning the feathers, etc.) were classified in one category.

## Vigilance vs. group size and composition

Proportion of records with a bird in vigilance posture (number of vigilance records / number of all 15-second interval records) is used as an index of vigilance activity of an individual. To avoid pseudo-replication due to sampling of several individuals from the same group, an average vigilance index was calculated for each group only with one individual per a group. The proportions of vigilance (*vigilance index*) were transformed using the Freeman-Tukey transformation (Zar 2013); designed to normalize the data and to reduce the heteroscedacity for the parametric analysis of regression and correlation. Regression analysis was performed to study the relationship between number of birds and the Freeman-Tukey transformed vigilance index. Stepwise backward selection of the regression model (Statsoft Inc 1999) was applied to analyze if the numbers of the Red-crowned, White-naped, or both crane species statistically correlate with the vigilance of the White-naped cranes in two-species flocks.

## Results

**Figure 1.**
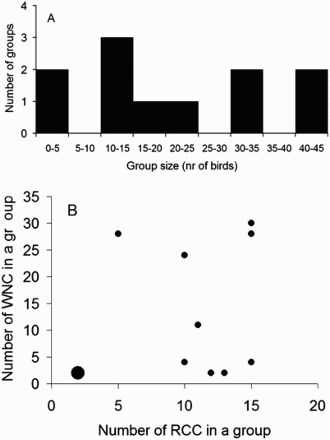
Group size distribution among the two-species flocks of cranes used in this study (**a**) and relationship between the number of Red-crowned cranes (RCC) and the White-naped cranes (WNC) in a group (**b**). The larger diameter circle in **b** indicates that two groups had the same values.

**Figure 2.**
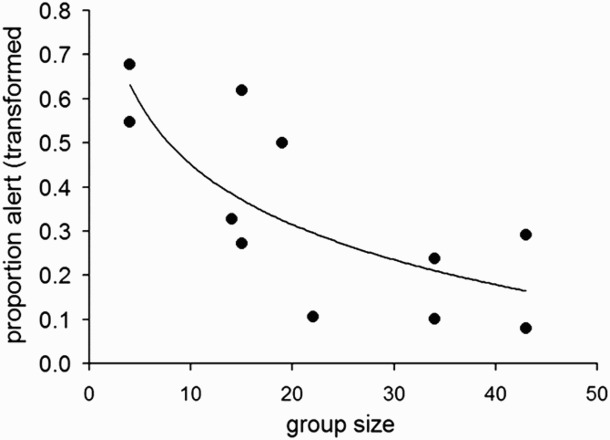
Relationship between vigilance of the White-naped cranes and the total number of birds in a group (nr of White-naped cranes + nr of Red-crowned cranes) in two-species groups of cranes in the Demilitarized Zone, Korea. (correlation: *r* = 0.74, *n* = 11 groups, *p *= 0.0094). The logarithmic fit: *y *= −0.45*ln(*x*) +0.91*; R *= 0.77, *N *= 11, *p* = 0.006.

**Figure 3.**
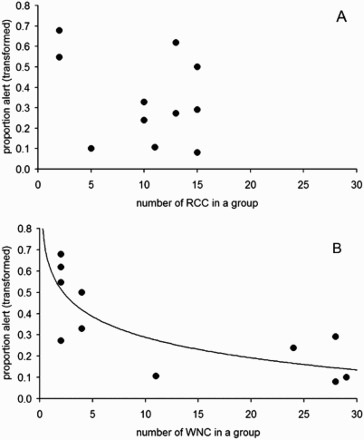
Relationship between vigilance of the White-naped cranes and the number of conspecifics (**a**) and the Red-crowned cranes, (**b**) in two-species groups of cranes in the Demilitarized Zone, Korea. The logarithmic fit in **a**: *y *= −0.14*ln(*x*) +0.61; *r *= −0.79, *n *= 11, *P *< 0.005.

The groups comprised ranging 4–43; including 2–15 Red-crowned cranes and 2–28 White-naped cranes totaling 11 different groups (Figure 1). There was a negative relationship between White-naped crane's vigilance and the total number of cranes in a group (no. of White-naped cranes + no. of Red-crowned cranes; Figure 2.; *r *= 0.74, *n *= 11 groups, *p *= 0.009). Logarithmic regression, predicted by standard vigilance models, also fits the results well (*r *= 0.77, *n *= 11, *p *= 0.006; *y *= −0.45*ln(*x*) +0.91). Because total group size is a sum of Red-crowned crane and White-naped crane it is not surprising that both variables, the number of Red-crowned crane and the number of White-naped crane, were positively correlated with the total group size (White-naped crane significantly: *r *= 0.94, *n *= 11, *p *= 0.00002; Red-crowned crane marginally non-significantly: *r *= 0.54, *n *= 11, *p *= 0.084). However, there was no significant correlation between the number of White-naped crane and the number of Red-crowned crane in a group (Figure 1(b); *r *= 0.22, *n *= 11, *p *= 0.51). Therefore we treated the two variables, the number of White-naped crane and the number of Red-crowned crane in a group, as independent factors that may affect vigilance of White-naped crane. If the negative relationship between total group size and White-naped crane's vigilance is caused by an increase in the number of White-naped crane as well as Red-crowned crane, we expect a decrease in vigilance in response to an increase in the number of one of the two species while statistically correcting (in multiple regression) for the effect of the number of the other species. When the numbers of both species were included as independent variables in the regression analysis (*r *= −0.74, *n *= 11; *p *= 0.040) the effect of the number of conspecific cranes (*t*-test for regression coefficients *t*_8 _= −2.82, *p *< 0.023), but not the Red-crowned cranes (*t*_8 _= −0.73, *p *> 0.481), on vigilance was significant (regression line: *y *= −0.012*x_WNC_* −0.008*x_RCC_* + 0.569; where *x_WNC_* and *x_RCC_* indicate the numbers of the White-naped and the Red-crowned cranes respectively). Backward selection method (Statsoft Inc 1999) resulted in a regression model with the effect of the number of Red-crowned cranes (Figure 3(A)), and the White-naped cranes (Figure 3(B)), on the vigilance of White-naped cranes in two-species groups (*r *= −0.72, *n *= 11; *p = *0.012; regression line: *y *= −0.01x + 0.50; logarithmic regression fit: *r *= −0.79, *n *= 11, *p *< 0.005; *y *= −0.14*ln(*x*) +0.61). The figures also indicated that the dominant of Red-crowned cranes usually form small number of individuals in a group (< 15 individuals) whereas subordinate White-naped cranes form large number of individuals (up to 30). This indicated that aggressive behavior of dominant species usually form smaller group than the subordinate species.

Associations of three or four White-naped cranes may comprise families of two parents and one or two young birds. Could higher vigilance in mixed groups with smaller number of conspecifics reflect the need for an especially increased vigilance to protect relatives (offspring protection hypothesis)? We reject this hypothesis as the main cause for the negative relationship in Figure 3(A) because the White-naped crane's vigilance remained significantly negatively affected by the number of White-naped cranes (linear regression *P *< 0.03; logarithmic regression *P *< 0.02) after the two mixed-species groups with families (each comprising two adults and two young) of White-naped cranes were removed from the analysis. To remove a potential bias due to differences in the observed range of values of the independent variables (the numbers of individuals per group were 2–15 for Red-crowned crane and 1–28 for White-naped crane) we calculated regressions excluding groups where the number of the White-naped cranes was larger than the maximal number of the Red-crowned cranes observed during this study (i.e. larger than 15 birds). After this correction, the vigilance of White-naped cranes also depended on the number of conspecifics (*r *= −0.75, *n *= 7, *p = *0.05) rather than the heterospecifics (*r *= −0.42, *n *= 7, *p > *0.34) in a group. Thus, the White-naped cranes decreased their vigilance when the total number of individuals in a group increased, and this appeared to be caused by the associated increase in the number of conspecifics, but not heterospecifics, in a group.

## Discussion

Cranes are vigilant due to a variety of factors (Levy 2000; Frid and Dill 2002; Aviles and Bednekoff 2007) including the presence of large birds of prey and human disturbance at the study site (Yoo 2004; Wang et al. 2011). Therefore one function of vigilance of cranes at our study site appears to be early detection of an approach of a predator (danger). Hence, the negative relationship between the group size and the White-naped crane's vigilance, as well as between the number of conspecifics and the White-naped crane's vigilance, together with the positive correlation between group size and the number of the White-naped cranes, are consistent with the *antipredatory vigilance* hypothesis which predicts decrease in antipredatory vigilance as the chances of predator detection by a group increase (Pulliam 1973).

The number of White-naped cranes and Red-crowned cranes in a group were not correlated. Therefore we were able test their independent effects on the White-naped crane's vigilance avoiding problems related to colinearity of independent variables. The results suggested that, although presence of the Red-crowned cranes decreases the average level of vigilance in comparison to the vigilance in mono-specific flocks (Lee et al. 2007a), the White-naped cranes do not respond to changes in the number of the Red-crowned cranes in two-species groups. This lack of relationship between the number of the Red-crowned cranes and the White-naped crane's vigilance, despite the marginally non-significant positive relationship between the number of the Red-crowned cranes and the total group size, appears inconsistent with the classical antipredatory hypothesis (Pulliam 1973). This hypothesis predicts lower vigilance as the number of the Red-crowned cranes increases. One possible explanation is that the White-naped cranes respond to the total groups size, but since the correlation between the total group size and the number of heterospecifics appears weaker than with the number of conspecifics (see results section) this leads to the lack of significant association between the number of heterospecifics and the White-naped crane's vigilance. However, given the presence of marginally non-significant and clearly positive association between the number of Red-crowned cranes and total group size, the non-significant effect of the number of the Red-crowned cranes on the White-naped crane's vigilance calls for another explanation. We propose that these results are consistent with the ‘*anti-dominant’ vigilance* hypothesis, and that the White-naped cranes’ vigilance to detect approaching Red-crowned cranes cancels out the otherwise expected negative correlation between the number of Red-crowned cranes and the White-naped crane's antipredatory vigilance. Similar effects of the number of scrounging dominants have already been suggested in mono-specific (Knight and Knight 1986) and mixed-species (Lee and Jablonski 2000) flocks of birds.

In summary, this is one of only a few reports (Lazarus and Symonds 1992; Metcalfe 1984; Thompson and Barnard 1983) that examine vigilance in mixed-species flocks of birds as a function of the numbers, rather than only presence or absence, of heterospecific as well as conspecific individuals in a group in one of the most important winter refuges. The correlational evidence presented here suggests that, in accordance with the *antipredatory vigilance* hypothesis the vigilance of a subordinate species correlates negatively with group size, and that this appears to be due to a negative correlation between vigilance and the number of conspecifics in a group. Lack of such negative correlation between vigilance and the number of dominant heterospecifics in a group, despite the marginally significant positive association between total group size and the number of dominants, is consistent with the *anti-dominant vigilance* hypothesis. According to this hypothesis, the benefits of decreased individual vigilance associated with flock formation may be offset by the presence of dominant species, which scrounges from the subordinate species and causes increase in its vigilance.

Due to the Allee effect, local population size can be an important factor in species fitness (Stephens and Sutherland 1999), particularly in species that depend on group activities for vigilance, foraging and protection. Further, because interspecific and intraspecific behavioral interactions may affect avian extinctions and endangerments (Reed 1999), the findings presented here may help in managing winter refuges for the two crane species. Previous research has investigated potential management strategies for crane conservation in this region (Lee et al. 2007b) and concerted efforts are needed to ensure their continued presence and success.
